# Identification of Drug Repurposing Opportunities of Immunomodulatory Drugs for Inflammatory Bowel Disease Through Inverse Pharmacovigilance Signal Detection in the FAERS Database

**DOI:** 10.3390/jcm15062172

**Published:** 2026-03-12

**Authors:** Katarina Đogatović, Katarina Vučićević, Milena Kovačević, Milica Ćulafić, Branislava Miljković, Sandra Vezmar Kovačević

**Affiliations:** Department of Pharmacokinetics and Clinical Pharmacy, Faculty of Pharmacy, University of Belgrade, 11000 Belgrade, Serbia

**Keywords:** drug repurposing, pharmacovigilance, signal detection, data mining

## Abstract

**Background/Objectives:** Drug repurposing represents a promising strategy to expand therapeutic options for inflammatory bowel disease (IBD), a chronic condition with persistent unmet clinical needs. This study aimed to identify existing drugs with potential relevance for IBD by exploring inverse associations in the FDA Adverse Event Reporting System (FAERS) as a hypothesis-generating, real-world data approach. **Methods:** In this retrospective observational pharmacovigilance study, drug–IBD associations were extracted from the FAERS database using OpenVigil 2.1. Inverse associations were identified based on reporting odds ratios (ROR) < 1 with adjusted *p*-values < 0.05. Identified drug–event pairs were further evaluated for pharmacokinetic feasibility, clinical applicability, and biological plausibility in the context of IBD, with the exclusion of drugs with implausible indications, contraindications, or mechanisms inconsistent with IBD pathophysiology. Given the immune-mediated nature of IBD and the breadth of the identified candidates, detailed evaluation focused on immunomodulatory agents. **Results:** Among the 3585 initial drug–IBD combinations, 73 candidates met the predefined criteria for statistical significance and feasibility. From these, nine drugs were prioritized based on inverse signal strength and mechanistic relevance to immune modulation pathways implicated in IBD. The strongest inverse association with IBD was observed for lenalidomide (ROR 0.056, 95% CI 0.043–0.073), followed by dupilumab (ROR 0.213, 95% CI 0.185–0.245), cyclophosphamide (ROR 0.215, 95% CI 0.175–0.265), fingolimod (ROR 0.216, 95% CI 0.205–0.334), dimethyl fumarate (ROR 0.332, 95% CI 0.275–0.400), apremilast (ROR 0.357, 95% CI 0.296–0.431), imatinib (ROR 0.423, 95% CI 0.339–0.527), glatiramer acetate (ROR 0.446, 95% CI 0.352–0.565), and interferon beta-1a (ROR 0.594, 95% CI 0.533–0.662). These agents possess immunomodulatory properties relevant to inflammatory pathways implicated in IBD; however, clinical evidence supporting the therapeutic efficacy of some candidates remains variable or incomplete. **Conclusions:** By integrating inverse signal detection with clinical and biological assessment, this study demonstrates how pharmacovigilance data can be extended from traditional safety surveillance toward systematic drug repurposing applications. The findings generate testable hypotheses and highlight candidate therapies that warrant further experimental and clinical investigation in IBD.

## 1. Introduction

Inflammatory bowel disease (IBD), which includes Crohn’s disease (CD) and ulcerative colitis (UC), presents a significant global health issue, with significant variations in incidence and prevalence across different regions. Annual incidence rate estimates range from 10.5 to 46.14 per 100,000 in Europe, 1.37 to 1.5 per 100,000 in Asia and the Middle East, 23.67 to 39.8 per 100,000 in Oceania, 0.21 to 3.67 per 100,000 in South America, and 7.3 to 30.2 per 100,000 in North America [1]. Furthermore, a study analyzing trends from 1990 to 2019 reported that the global incidence of IBD has risen, and emphasized a shift in the epidemiology of IBD, with significant increases observed in regions undergoing rapid socioeconomic development in contrast to its historically higher prevalence in Western nations [2]. IBD develops due to a combination of genetic factors, environmental triggers, and immune system dysfunction [3]. A key aspect of IBD is a weakened intestinal barrier that allows microbial antigens to trigger an exaggerated immune response, driven by pro-inflammatory cytokines like Tumor necrosis factor-alpha (TNF-α), Interleukin-12 (IL-12) and Interleukin-23 (IL-23) [4]. An imbalance in gut microbiota (dysbiosis) further fuels inflammation, leading to chronic tissue damage and complications [5]. While current treatments aim to control inflammation, manage flare-ups, and prevent relapses, challenges remain—particularly the lack of oral treatment options [6]. Additionally, a substantial proportion of patients experience primary non-response, secondary loss of response, or treatment-limiting adverse effects, highlighting the need for novel therapeutic strategies.

Drug repurposing has emerged as a promising approach to accelerate the discovery of new IBD treatments by identifying existing drugs with known safety profiles that may have therapeutic potential [7]. This approach can substantially reduce development time, cost, and early-stage safety uncertainties compared with de novo drug development. Real-world pharmacovigilance data from post-marketing surveillance (PMS) systems such as the FDA Adverse Event Reporting System (FAERS) and VigiBase provide a valuable resource for identifying unexpected drug-disease associations. In particular, inverse signal detection—where drugs are reported less frequently in association with a disease than expected—may suggest potential protective or disease-modifying effects worthy of further investigation [8]. Given the targeted action of immunomodulators on key inflammatory pathways and success in treating other immune-related diseases, these drugs represent strong candidates for further investigation [9]. The aim of this study was to mine global pharmacovigilance data for potential drug repurposing candidates in IBD, with a particular focus on immunomodulatory agents, including monoclonal antibodies.

## 2. Materials and Methods

Source data was retrieved and analyzed using the OpenVigil 2.1 [10] and MedDRA-v24 (data 2003Q4 to 2024Q3). The Medical Dictionary for Regulatory Activities (MedDRA) is a clinically validated international medical terminology dictionary-thesaurus, organized in a hierarchical structure of different terms [11]. An observational, retrospective pharmacovigilance case/non-case study in FAERS using OpenVigil 2.1 and scanned for inverse signals of existing drugs and events of interest, retrieved as cases related to the PTs (preferred terms) of the MedDRA (version 24.0). The chosen algorithm was the computation of the Reporting Odds Ratio (ROR, calculated similarly to the odds ratio in case–control studies) to estimate the extent to which a given ADR is associated with a specific drug, relative to patients using reference drugs [12]. The ROR, 95% confidence interval (95% CI) and adjusted *p*-value (statistically significant if padj < 0.05), were calculated [13]. An ROR > 1 suggests that the ADR is reported more frequently for the drug of interest compared to other drugs in the database. Conversely, an ROR < 1 indicates that the drug-adverse event combination is less frequent than expected, potentially suggesting an unexpected protective effect. During the data mining process, MedDRA terms of “Ulcerative colitis” or “Crohn’s disease” were selected as search criteria. This selection was based on the understanding that both conditions, although distinct, fall under the broader category of IBD and share similarities in terms of symptoms and pathological mechanisms. A specific role of a drug in the drug–event relationship (i.e., suspect/concomitants) was not predefined to ensure a broader background case set for each drug. This decision reflects the exploratory nature of inverse signal detection and acknowledges that drug role assignment in FAERS is reporter-dependent and not standardized. Benjamini–Hochberg correction was applied to the *p*-values to control the false discovery rate. Deduplication, mapping and initial cleaning of drug names were performed using the automated normalization pipeline implemented in OpenVigil 2, which harmonizes verbatim entries against curated reference sources including DrugBank and Drugs@FDA. This process standardizes brand names, salt forms, and formulation variants to their corresponding active substances and excludes reports with drug entries that cannot be unambiguously resolved. Combination products were decomposed into individual active substances, and duplicate reports were managed using FDA-recommended case version control logic. Following this automated step, manual curation was performed. Clinical feasibility filtering was predefined and limited to the exclusion of non-systemic formulations, diagnostic agents, withdrawn products, pharmacokinetically implausible compounds, and drugs with established lack of efficacy or documented disease-aggravating effects in IBD. These exclusions were applied to enhance interpretability and clinical coherence and were not based on observed signal strength. No mechanistic plausibility filtering was applied during statistical screening. All drugs meeting the predefined disproportionality thresholds, technical, and feasibility criteria were retained. Mechanistic considerations were explored only after signal identification to contextualize findings.

A conservative minimum case threshold of ≥40 reports (*n* > 39 in the dataset query) was predefined to reduce instability associated with sparse data. While typical safety signal detection may apply lower minimum case counts (e.g., ≥3–5 reports), higher thresholds are frequently used in exploratory pharmacovigilance and repurposing research to enhance robustness. Given the susceptibility of inverse signals to small-number bias, this study prioritized specificity and estimate stability over maximal sensitivity.

Following statistical screening, drugs were categorized by therapeutic class. For the present manuscript, immunomodulatory drugs were prioritized for in-depth analysis due to the immune-mediated pathophysiology of IBD.

The inverse signal screening generated candidates across multiple therapeutic classes. For the present analysis, a structured literature review was performed to assess available preclinical and clinical evidence for a selected subset of candidate drugs, with a focus on drugs primarily indicated for immune-mediated diseases, particularly monoclonal antibodies and immunomodulatory agents, to allow for a coherent mechanistic evaluation within the immunopathological framework of IBD. Preclinical evidence was defined as experimental data derived from in vitro or animal models relevant to intestinal inflammation or immune modulation. Clinical evidence was defined as data from human studies, including randomized controlled trials, observational studies, or case reports in IBD or related inflammatory conditions. Evidence was categorized descriptively and was not used as a statistical inclusion or exclusion criterion.

Ethics committee approval was not required as the data source is publicly available and fully anonymized.

## 3. Results

**Figure 1:**
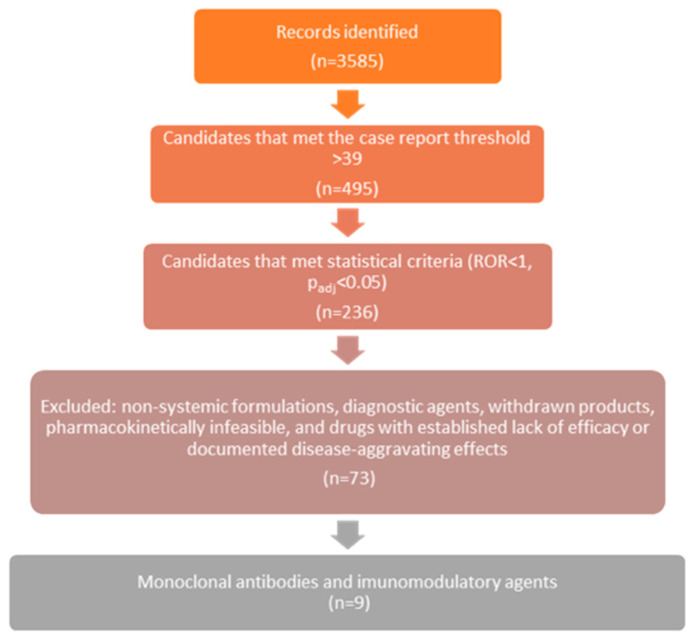
Flow diagram of the drug candidate selection process.

The selection process for potential drug candidates is visually represented in Figure 1, which illustrates the stepwise refinement from an initial 3585 drug–event combinations to the final 73 candidates. This flow diagram outlines the application of inclusion and exclusion criteria, including statistical thresholds, pharmacokinetic feasibility, and clinical relevance, ensuring a rigorous and systematic approach to candidate identification.

The inverse signals were further analyzed to generate hypotheses regarding potential new drug associations. Table 1 presents all 73 drug candidates that met the previously described selection criteria, including statistical significance, pharmacokinetic feasibility, and clinical relevance.

**Figure 2:**
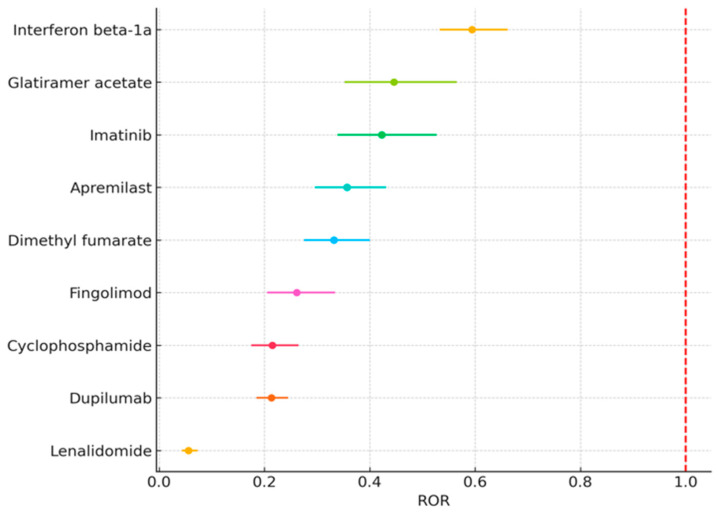
Forest plot of Reporting Odds Ratios (RORs) with 95% confidence intervals (CIs) of the selected candidate drugs.

The list was further refined to include drugs primarily indicated for immune-mediated diseases or conditions involving dysregulated immune responses, with a focus on monoclonal antibodies and immunomodulatory agents. This selection resulted in nine candidates. Figure 2 provides a forest plot of these nine selected candidates, displaying their respective confidence intervals to illustrate the strength and direction of their associations.

## 4. Discussion

Our study introduces a novel approach to identifying potential drug candidates for repurposing in IBD treatment. The present work focused on immunomodulatory drugs identified through inverse signal detection. While additional therapeutic classes demonstrated statistically significant inverse associations, a comprehensive mechanistic evaluation of all candidates was beyond the scope of this manuscript.

Among the nine prioritized candidates, four demonstrated preclinical evidence without completed clinical trials (fingolimod, dimethyl fumarate, glatiramer acetate, imatinib), three had mixed or inconclusive clinical data (lenalidomide, apremilast, interferon beta-1a), one showed clinical activity primarily in refractory disease settings (cyclophosphamide), and one is currently undergoing large-scale clinical evaluation (dupilumab). The variability in supporting evidence highlights the heterogeneity in translational maturity across candidates and reinforces the hypothesis-generating nature of inverse signal detection.

1. Immunomodulators and targeted therapies

While initially utilized in cancer treatment, their unintended impact on the immune system has been leveraged to address non-malignant diseases where autoimmunity plays a significant role in the disease process. In a study that investigated the therapeutic effects of lenalidomide and pomalidomide in experimental models of acute colitis, which mimic the features of human CD and UC, lenalidomide treatment resulted in full protection against IBD. The lenalidomide-treated mice exhibited rapid recovery of body weight loss, improved survival rates, reduced colon damage and inflammation, and restoration of healthy appearance [14]. A randomized, double-blind, placebo-controlled trial of lenalidomide in the treatment of moderately severe active CD evaluated the efficacy and safety of lenalidomide at daily doses of 5 mg and 25 mg compared to the placebo in patients with moderate-to-severe CD [15]. The clinical remission rates in both lenalidomide groups were not significantly different from those in the placebo group. The study design, including continuation of background corticosteroid therapy, may have influenced the observed outcomes, as corticosteroids can modulate immune responses and potentially alter the pharmacodynamic effects of immunomodulatory agents [16]. Additionally, higher-dose combination therapy was associated with increased adverse events and treatment discontinuation. While preclinical models of colitis have suggested potential anti-inflammatory effects of lenalidomide [14], the divergence between the experimental findings and clinical trial outcomes highlights the complexity of translating immunomodulatory mechanisms into therapeutic efficacy. The strong inverse signal observed in FAERS should therefore be interpreted cautiously and viewed as hypothesis-generating rather than confirmatory of clinical benefit. Further well-designed studies would be required to clarify the therapeutic potential and safety profile of lenalidomide in IBD. Dupilumab’s mechanism in blocking interleukin-4/interleukin-13 (IL-4/IL-13) pathways suggests that it may address specific inflammatory processes in IBD, offering hope for a broader application beyond its current use for atopic diseases and eosinophilic esophagitis. IL-4’s role in IBD pathogenesis is supported by genetic associations, experimental models, and its increased production in colitis, with dual IL-4/IL-13 inhibition showing therapeutic benefits [17]. A large-scale, ongoing clinical trial has been enrolling participants worldwide, with criteria focusing on moderately to severely active UC. Early data suggest potential for symptom reduction and histologic remission in patients who are unresponsive to corticosteroids and other biologic treatments [18]. A review of sphingosine 1-phosphate (S1P) inhibitors for treating IBD provides a concise summary of the current clinical evidence of efficacy of S1P inhibitors, and explores fingolimod’s success in preclinical studies, as currently there are no clinical studies registered testing fingolimod in IBD [19]. Dimethyl fumarate (DMF) has shown immunomodulatory, antioxidant, and anti-inflammatory effects. Research highlights DMF’s role in regulating critical inflammatory pathways, particularly by activating nuclear factor erythroid 2-related factor 2 (Nrf2). Nrf2 activation is associated with reducing oxidative stress in the gut and promoting gut homeostasis, which is essential for controlling inflammation in IBD [20]. A study on dextran sulfate sodium (DSS)-induced colitis in mice showed that DMF provided dose-dependent protection by minimizing body weight loss, maintaining colon length, and reducing colonic damage. DMF demonstrated a strong antioxidant potential, suppressed the expression of pro-inflammatory cytokines such as Interleukin-1-beta (IL-1β), TNF-α, and Interleukin-6 (IL-6) at both the protein and mRNA levels, and a reduced caspase-1 activation in DMF-treated mice further suggested the inhibition of NOD-, LRR- and pyrin domain-containing protein 3 (NLRP3) inflammasome activation [21].

2. Cytotoxic agents

Cyclophosphamide suppresses the immune system through DNA alkylation, leading to the apoptosis of immune cells that drive inflammation, and has shown promise as a treatment for severe, steroid-refractory IBD, particularly through intravenous pulse therapy [22]. Studies have demonstrated its ability to induce remission in patients unresponsive to standard treatments, with some achieving long-lasting remission after just a few cycles [23]. Additionally, its use in severe CD suggests that it may be a viable induction therapy when other options fail, highlighting its potential role in refractory cases [24].

3. Small molecule inhibitors

Apremilast, an oral phosphodiesterase 4 (PDE4) inhibitor, modulates inflammatory responses by reducing the production of pro-inflammatory cytokines such as TNF-α, IL-1β, and IL-6. A double-blind, phase 2 trial evaluated apremilast in adults with active UC over 52 weeks, showing that the 30 mg dose led to higher rates of clinical remission at 12 weeks (31.6% vs. 12.1% with placebo, *p* = 0.01) and maintained remission in 40.4% at week 52. While the primary endpoint was not met, apremilast demonstrated improvements in clinical, endoscopic, and inflammatory markers, with headache and nausea as common adverse events [25]. In a series of six patients with refractory CD treated with imatinib, four achieved clinical and endoscopic remission within a median of three months. These remissions were sustained for periods ranging from nine months to seven years, suggesting that imatinib may be a viable therapeutic option for refractory CD [26]. Additionally, in a rat model of acetic acid-induced UC, Imatinib pretreatment significantly reduced colonic damage, inflammation, oxidative stress, and altered inflammatory signaling pathways, namely nuclear factor kappa-light-chain-enhancer of activated B cells (NF-κB), Janus kinase 2/Signal transducer and activator of transcription 3 (JAK2/STAT3), and Cyclooxygenase-2 (COX2), suggesting its potential as a therapeutic option for UC [27].

4. Biologics and immunotherapies

Following evidence of the therapeutic efficacy of glatiramer acetate in various IBD animal models [28,29], a case report detailing the first successful induction of remission in a patient with active CD using glatiramer acetate provides optimism for further exploration and investigation [30]. In a randomized, placebo-controlled, dose-escalating study, Interferon beta-1a (IFN-β-1a) led to a desired clinical response in 50% of patients in the group compared to 14% in the placebo group (*p* = 0.14). Remission was achieved in three patients in the IFN-β-1a group, while none achieved remission in the placebo group (*p* = 0.02), and a very favorable safety profile was noted [31]. Another pilot study demonstrated a high remission rate of 88% during induction therapy [32]. A randomized, double-blind, placebo-controlled trial reported that IFN-β-1a therapy, while safe, did not prove effective for steroid-refractory UC, possibly due to insufficient dosing, a brief treatment duration, or a high dropout rate [33].

The translational maturity of the identified candidates is heterogeneous, with some supported primarily by preclinical data and others having preliminary or mixed clinical evidence. A structured overview of the mechanistic rationale and available preclinical and clinical evidence is provided in Table 2.

This study was based on a spontaneous reporting system inherently subject to underreporting, reporting bias, confounding by indication, and heterogeneity in reporting practices across countries. As such, inverse disproportionality signals may reflect prescribing patterns, comorbidity structures, or differential reporting behaviors rather than true protective biological effects. The database lacks detailed clinical covariates, precluding adjustment for potential confounders. Individual case medical histories and concomitant medications were not systematically reviewed, and causality assessments, case narratives, time-to-onset data, treatment duration, and dechallenge/rechallenge information were not consistently available. Without temporal context, it is not possible to determine whether drug exposure preceded disease onset, modified disease activity, or was unrelated to the reported outcome. Consequently, the findings should be interpreted as hypothesis-generating associations rather than evidence of clinical efficacy. This analysis provides a foundation for further mechanistic research and controlled epidemiological or clinical studies to validate the efficacy and safety of the identified candidates.

Several drugs identified with inverse signals (e.g., statins, antihypertensives, benzodiazepines) are commonly prescribed in older populations with metabolic or cardiovascular comorbidities. Their inverse disproportionality may therefore reflect demographic structure, comorbidity clustering, or channeling bias rather than a true protective biological effect on IBD. Spontaneous reporting systems such as FAERS do not provide reliable exposure denominators, and age, sex, and indication data are frequently incomplete. Consequently, confounding by indication, co-medication patterns, and demographic-related reporting biases cannot be fully controlled in this framework. Importantly, inverse signal detection reflects statistical reporting patterns rather than confirmed pharmacologic protection. Furthermore, polypharmacy is common in FAERS reports, and co-medication patterns may influence disproportionality estimates. Because FAERS does not provide reliable denominators or comprehensive clinical covariates, adjustment for co-medication frequency was not feasible within this framework. Therefore, inverse signals may reflect complex prescribing patterns rather than independent pharmacologic effects.

Restricting the analysis to “suspect” drugs may be appropriate in traditional safety signal detection aimed at identifying potential causal associations. However, in inverse signal detection, such restriction may introduce bias, as potentially protective agents are unlikely to be labeled as causative in spontaneous reports. Inclusion of all drug roles therefore better aligns with the exploratory and associative nature of inverse disproportionality analysis. Although incorporating concomitant drugs may introduce additional noise due to non-causal co-reporting, limiting the analysis to “suspect” drugs could disproportionately exclude chronic background therapies and distort exposure patterns in immune-mediated diseases. Additionally, the inclusion of concomitant medications may introduce distortion if certain drugs are routinely co-prescribed with agents known to cause gastrointestinal adverse events, potentially influencing reporting patterns independent of a true protective effect. Such findings may therefore reflect prescribing practices rather than genuine disease modification. Accordingly, the findings should be interpreted as associative signals rather than evidence of causality.

It is important to emphasize that inverse signal detection does not establish clinical efficacy; rather, it generates hypotheses that require validation through controlled mechanistic and clinical studies, particularly where prior trial results have been mixed or negative. These findings should therefore be interpreted as hypothesis-generating and require validation in pharmacoepidemiologic or mechanistic studies.

Future investigations into the mechanisms of action and clinical outcomes of these candidates will advance personalized and targeted therapies for IBD. By employing a systematic, data-driven methodology, our approach leverages real-world evidence from global adverse event reports to uncover potential treatments with established safety profiles. This method offers a faster, more resource-efficient alternative to traditional drug development, reducing the risks and costs of creating entirely new drugs.

Beyond IBD, the application of this methodology represents a significant advancement in the field of drug repurposing, providing a scalable and innovative framework for identifying treatments across various conditions.

## 5. Conclusions

Evidence supporting the repurposing potential of the identified candidates for IBD varies across agents. While several drugs are supported by promising preclinical or early clinical findings, others have only limited mechanistic or experimental evidence. The variability in supporting evidence highlights the heterogeneity in translational maturity across candidates and reinforces the hypothesis-generating nature of inverse signal detection. At the same time, these findings identify important knowledge gaps and provide opportunities for further research to clarify mechanisms of action and evaluate the therapeutic potential of these agents in IBD.

## Figures and Tables

**Table 1:** Selected 73 drug repurposing candidates, in ascending order of ROR with 95% confidence interval.

Drug	ROR	ROR Lower Bound	ROR Upper Bound
Lenalidomide	0.056	0.043	0.073
Risperidone	0.129	0.096	0.173
Levetiracetam	0.170	0.129	0.225
Dulaglutide	0.181	0.136	0.242
Insulin lispro	0.206	0.161	0.263
Varenicline	0.207	0.16	0.268
Dupilumab	0.213	0.185	0.245
Cyclophosphamide	0.215	0.175	0.265
Clozapine	0.225	0.182	0.280
Valsartan	0.240	0.206	0.280
Insulin glargine	0.246	0.205	0.295
Fingolimod	0.261	0.205	0.334
Lamotrigine	0.262	0.211	0.326
Rivaroxaban	0.287	0.242	0.341
Carvedilol	0.297	0.235	0.375
Carbamazepine	0.313	0.236	0.414
Aripiprazole	0.318	0.263	0.386
Enoxaparin	0.319	0.239	0.424
Dimethyl fumarate	0.332	0.275	0.400
Atorvastatin calcium	0.334	0.262	0.425
Insulin	0.340	0.295	0.390
Apixaban	0.348	0.305	0.396
Insulin aspart	0.349	0.267	0.455
Apremilast	0.357	0.296	0.431
Lisinopril	0.364	0.318	0.416
Somatotropin	0.373	0.279	0.498
Clopidogrel	0.385	0.329	0.452
Simvastatin	0.394	0.343	0.452
Empagliflozin	0.400	0.311	0.514
Warfarin	0.415	0.359	0.479
Diazepam	0.418	0.343	0.510
Fenofibrate	0.418	0.307	0.567
Liraglutide	0.419	0.319	0.552
Nifedipine	0.419	0.311	0.566
Imatinib	0.423	0.339	0.527
Enalapril	0.430	0.325	0.567
Fexofenadine	0.435	0.346	0.545
Spironolactone	0.442	0.364	0.537
Metformin	0.446	0.407	0.489
Glatiramer acetate	0.446	0.352	0.565
Hydroxyzine	0.454	0.346	0.594
Sitagliptin	0.460	0.376	0.563
Clonidine	0.465	0.355	0.609
Metoclopramide	0.472	0.377	0.592
Pravastatin	0.478	0.381	0.600
Losartan	0.488	0.422	0.565
Pregabalin	0.496	0.442	0.557
Naltrexone	0.499	0.370	0.673
Verapamil	0.501	0.372	0.673
Alprazolam	0.532	0.463	0.610
Metoprolol	0.552	0.497	0.612
Topiramate	0.566	0.472	0.680
Rosuvastatin calcium	0.570	0.427	0.761
Fluconazole	0.586	0.457	0.753
Gabapentin	0.591	0.535	0.654
Interferon beta-1a	0.594	0.533	0.662
Nitroglycerin	0.597	0.459	0.776
Octreotide	0.599	0.458	0.782
Atorvastatin	0.603	0.551	0.661
Quetiapine	0.606	0.539	0.681
Irbesartan	0.613	0.482	0.779
Atenolol	0.618	0.526	0.726
Semaglutide	0.622	0.507	0.764
Diltiazem	0.624	0.514	0.758
Propranolol	0.624	0.509	0.764
Allopurinol	0.638	0.546	0.745
Amlodipine	0.641	0.589	0.698
Melatonin	0.648	0.480	0.874
Temazepam	0.694	0.512	0.939
Clonazepam	0.745	0.658	0.844
Famotidine	0.780	0.655	0.930
Venlafaxine	0.852	0.757	0.960
Bisoprolol	0.856	0.754	0.972

**Table 2:** Summary of the mechanistic rationale and preclinical/clinical evidence for prioritized candidates.

Drug	Mechanistic Relevance	Preclinical Evidence	Clinical Evidence	Level of Evidence
**Lenalidomide**	Immunomodulatory, anti-angiogenic	Protective effects in experimental colitis models	Randomized controlled trial negative; no significant remission vs. placebo	Preclinical positive/clinical negative
**Dupilumab**	IL-4/IL-13 inhibitor, reducing inflammation	Mechanistic rationale supported by experimental models	Ongoing large-scale clinical trial; early data suggest potential benefit	Emerging clinical evidence
**Cyclophosphamide**	Alkylating agent, suppressing immune cells	No preclinical evidence reported	Clinical reports of remission in steroid-refractory IBD (small studies)	Clinical positive (non-RCT)
**Fingolimod**	S1P receptor modulator, prevents lymphocyte egress	Protective effects in multiple experimental colitis models (IL-10−/−, DSS, TNBS)	No registered clinical trials in IBD	Preclinical only
**Dimethyl fumarate (DMF)**	Nrf2 activator, anti-inflammatory	DSS mouse colitis model; antioxidant and anti-inflammatory effects	No clinical trials in IBD reported	Preclinical only
**Apremilast**	PDE4 inhibitor, reducing cytokine production	No preclinical evidence reported	Phase 2 trial: remission signal; primary endpoint not met	Clinical mixed
**Imatinib**	Tyrosine kinase inhibitor, anti-fibrotic	Rat colitis model showing reduced inflammation and signaling modulation	Small case series (*n* = 6); 4 remissions reported	Preclinical + limited clinical
**Glatiramer acetate**	Immunomodulatory, inducing regulatory T cells	Multiple IBD animal models showing benefit	Single case report	Preclinical + very limited clinical
**Interferon beta-1a**	Immunomodulatory, antiviral and anti-inflammatory	No preclinical evidence reported	Mixed results: pilot positive; larger RCT negative	Clinical mixed

## Data Availability

The FDA Adverse Event Reporting System (FAERS) database and source are freely available. OpenFDA is freely accessible at https://api.fda.gov/drug/event.json (accessed on 24 December 2025). OpenVigil FDA can be used or downloaded at http://openvigil.sourceforge.net (accessed on 24 December 2025).

## Abbreviations

The following abbreviations are used in this manuscript:

COX2	Cyclooxygenase-2
DMF	Dimethyl fumarate
DSS	Dextran sulfate sodium
FAERS	FDA Adverse Event Reporting System
IBD	Inflammatory bowel disease
IFN-β-1a	Interferon beta-1a
IL-1β	Interleukin-1-beta
IL-4	Interleukin-4
IL-6	Interleukin-6
IL-12	Interleukin-12
IL-13	Interleukin-13
IL-23	Interleukin-23
JAK2/STAT3	Janus kinase 2/Signal transducer and activator of transcription 3
MedDRA	Medical Dictionary for Regulatory Activities
NF-κB	Nuclear factor kappa-light-chain-enhancer of activated B cells
NLRP3	NOD-, LRR- and pyrin domain-containing protein 3
Nrf2	Nuclear factor erythroid 2-related factor 2
PDE4	Phosphodiesterase 4
PMS	Post-marketing surveillance
PT	Preferred term
ROR	Reporting odds ratio
RCT	Randomized Controlled Trial
S1P	Sphingosine 1-phosphate
TNF-α	Tumor necrosis factor-alpha
UC	Ulcerative colitis
